# Peptide Vaccine Combined Adjuvants Modulate Anti-tumor Effects of Radiation in Glioblastoma Mouse Model

**DOI:** 10.3389/fimmu.2020.01165

**Published:** 2020-06-11

**Authors:** Thi-Anh-Thuy Tran, Young-Hee Kim, Thi-Hoang-Oanh Duong, Shin Jung, In-Young Kim, Kyung-Sub Moon, Woo-Youl Jang, Hyun-Ju Lee, Je-Jung Lee, Tae-Young Jung

**Affiliations:** ^1^Brain Tumor Research Laboratory, Chonnam National University Medical School and Hwasun Hospital, Chonnam National University, Hwasun, South Korea; ^2^Department of Neurosurgery, Chonnam National University Medical School and Hwasun Hospital, Chonnam National University, Hwasun, South Korea; ^3^Research Center for Cancer Immunotherapy, Chonnam National University Medical School and Hwasun Hospital, Chonnam National University, Hwasun, South Korea; ^4^Department of Internal Medicine, Chonnam National University Medical School and Hwasun Hospital, Chonnam National University, Hwasun, South Korea

**Keywords:** radiation, branched multipeptide, PADRE, poly-ICLC, anti-PD1, glioblastoma

## Abstract

Glioblastoma, the most common aggressive cancer, has a poor prognosis. Among the current standard treatment strategies, radiation therapy is the most commonly recommended. However, it is often unsuccessful at completely eliminating the cancer from the brain. A combination of radiation with other treatment methods should therefore be considered. It has been reported that radiotherapy in combination with immunotherapy might show a synergistic effect; however, this still needs to be investigated. In the current study, a “branched multipeptide and peptide adjuvants [such as pan DR epitope (PADRE) and polyinosinic-polycytidylic acid—stabilized with polylysine and carboxymethylcellulose—(poly-ICLC)],” namely vaccine and anti-PD1, were used as components of immunotherapy to assist in the anti-tumor effects of radiotherapy against glioblastomas. With regard to experimental design, immunological characterization of GL261 cells was performed and the effects of radiation on this cell line were also evaluated. An intracranial GL261 mouse glioma model was established, and therapeutic effects were observed based on tumor size and survival time. The distribution of effector immune cells in the spleen, based on cytotoxic T lymphocyte (CTL) and natural killer (NK) cell function, was determined. The pro-inflammatory and anti-inflammatory cytokine production from re-stimulated splenocytes and single tumor cells were also evaluated. As GL261 cells demonstrated both immunological characteristics and radiation sensitivity, they were found to be promising candidates for testing this combination treatment. Combinatorial treatment with radiation, vaccine, and anti-PD1 prolonged mouse survival by delaying tumor growth. Although this combination treatment led to an increase in the functional activity of both CTLs and NK cells, as evidenced by the increased percentage of these cells in the spleen, there was a greater shift toward CTL rather than NK cell activity. Moreover, the released cytokines from re-stimulated splenocytes and single tumor cells also showed a shift toward the pro-inflammatory response. This study suggests that immunotherapy comprising a branched multipeptide plus PADRE, poly-ICLC, and anti-PD1 could potentially enhance the anti-tumor effects of radiotherapy in a glioblastoma mouse model.

## Introduction

Glioblastoma (GBM) is the most malignant tumor of the central nervous system and is associated with poor prognosis and low survival. The survival rates of GBM patients have not demonstrated notable improvements over the last few decades (1). Therefore, a combination of several treatment methods is essential to overcome this type of cancer. Radiation therapy (RT) is commonly used to treat GBM; ~60% of patients with solid tumors are administered radiation as part of their treatment (2). RT involves the breakdown of double-stranded DNA, thereby affecting cancer cell survival and proliferation. It also enhances immunological aspects, such as tumor antigen presentation and immunomodulation, by exposing tumor antigens and making them visible to the immune surveillance machinery (3, 4). Preclinical evidence suggests that RT can prime the immune system to enhance the efficiency of immunotherapy and that a combination of RT with immunotherapy is more effective than monotherapy (5).

Developments in the field of immunotherapy have recently provided new options for the treatment of GBM. Although the brain is an immunologically distinct organ, the immune microenvironment offers sufficient opportunities to promote immune cell responses and modify the “cold” tumor status of GBM (6). Although vaccination appears to be a promising treatment strategy for improving the clinical outcomes of GBM patients, no successful result has been reported in phase III clinical trials of vaccines against GBM to date; moreover, vaccine therapy faces many challenges. Combinations of different therapy methods, such as various vaccination strategies, vaccinations with immune checkpoint inhibitors, or surgical resection with chemotherapy, radiotherapy, and immunotherapy, are potential future directions for GBM treatment (7, 8).

To enhance the function of vaccines, immune adjuvants have also been developed. Immune adjuvants are defined as compounds that act to accelerate, prolong, and enhance the antigen-specific immune response, thereby allowing the use of smaller antigen doses and fewer immunizations (9). Among the available peptide vaccine adjuvants, pan DR epitope (PADRE) is a synthetic epitope-based vaccine adjuvant that is used as a T-helper peptide that induces Th1 cell polarization. PADRE is derived from HLA-DR epitopes and a tetanus toxin fragment. The PADRE peptide can bind to many different types of MHC-II alleles to boost immune responses, leading to the enhanced anti-tumor efficacy of vaccines (10, 11). Previous clinical trials reported that a polyinosinic-polycytidylic acid—stabilized with polylysine and carboxymethylcellulose—(poly-ICLC)-combined tumor antigen-specific vaccine is effective at achieving a higher therapeutic index (12). Poly-ICLC stimulates the Th1-polarizing dendritic cells and microglia-expressed toll-like receptor 3 (TLR3), resulting in the anti-tumor immune response. In addition, poly-ICLC serves as a simple and low-cost pathogen-associated molecular pattern (PAMP) that can trigger the immune response against solid cancers. Phase II clinical trials have been initiated for poly-ICLC (13).

Although anti-PD1 has been approved for the treatment of multiple cancer types, the effects of anti-PD1 monotherapy are still uncommon and unpredictable in GBM treatment. Only a small subset of patients have shown beneficial effects in response to anti-PD1 monotherapy; therefore, this requires further evaluation (14). However, the efficacy of immune checkpoint blockade has been demonstrated in combination with RT and a peptide-based vaccine. In particular, the combination of anti-PD1 and localized RT was shown to result in long-term survival in orthotopic GBM mouse models (15). Moreover, combinatorial treatment with peptide-based vaccines and immune checkpoint inhibitors was demonstrated to prolong the survival of tumor-bearing mice via enhanced vaccine-induced immune responses and tumor-infiltrating CD8^+^ T cell counts, leading to delayed tumor growth (16).

Therefore, in this study, we aimed to determine the role of immunotherapy in modulating the anti-tumor effects of RT against GBM. For immunotherapy, branched multipeptide constructs based on the epidermal growth factor receptor 2 (ErbB2) and Wilms tumor gene 1 (WT1) peptides were used to stimulate antigen-specific cytotoxic T lymphocytes (CTLs). A combination of this branched multipeptide with peptide adjuvants, such as PADRE and poly-ICLC, was considered a component of the vaccine. We found that this vaccine, in combination with or without anti-PD1, modulated the anti-tumor effects of RT in a mouse GBM model.

## Materials and Methods

### Animals and Cell Lines

Six- to eight- week-old female C57BL/6 mice (H2b, IAb) were purchased from Orient Bio (Iksan, Republic of Korea). Mice were raised under specific-pathogen-free conditions. All animal care, experiments, and euthanasia were performed after obtaining approval from the Chonnam National University Animal Research Committee.

Mouse glioblastoma cell lines (GL261: H2b and IAb, Gibco-BRL, Gaithersburg, MD, USA), and mouse lymphoma cell lines (YAC-1, ATCC, Rockville, MD, USA), sensitive to the cytotoxic activity of natural killer (NK) cells in mice, were used for cell culture. GL261 cells were maintained in Dulbecco's Modified Eagle's Medium (DMEM) and YAC-1 cells were grown in Roswell Park Memorial Institute (RPMI) 1640 medium supplemented with 10% fetal bovine serum (FBS) and 1% penicillin-streptomycin (P/S) at 37°C in an atmosphere of 5% CO_2_.

### Peptide Synthesis and Antibodies

All peptides were commercially synthesized by the Peptron Company (Daejeon, Republic of Korea) with a purity >95% as assessed by reverse phase high-performance liquid chromatography. The branched multipeptide was synthesized by incorporating two single peptides, mouse modified 9-mer WT1 peptide (H2b-restricted WT1_235−243_: CYTWNQMNL) and the mouse 9-mer epidermal growth factor receptor 2 peptide (H2b-restricted ErbB2_63−71_: TYLPANASL) (predicted binding scores from SYFPEITHI: http://www.syfpeithi.de). Mini-polyethylene glycol (mini-PEG) spacers were used to synthesize the corresponding branched multipeptide, which was designated as CYTWNQMNL-miniPEG2-K (TYLPANASL-miniPEG2) shown in Figure S1. A pan HLA-DR binding epitope (IAb-restricted PADRE, ak-Cha-VAAWTLKAAa-Z-C) was also synthesized (17). All peptides were dissolved in dimethyl sulfoxide (DMSO) and diluted with phosphate buffered saline (PBS). Mouse anti-PD1 (clone J43) was used for flow cytometry and mouse anti-PD1 (clone RMP1-14) was used for *in vivo* blockade. All antibodies were purchased from BioXcell (West Lebanon, NH, USA).

### Western Blotting

The expression of ErbB2, WT1, and programmed death ligand 1 (PDL1) in the GL261 cells before and after radiation was confirmed by western blotting. In general, the cells were exposed to 2, 4, or 6 Gy of radiation and cultured. The cells were harvested after the indicated time periods (0 and 24 h) for western blot analysis. The bicinchoninic acid (BCA) Protein Assay Kit (Thermo Scientific, USA) was used to measure protein concentration. Thereafter, SDS-PAGE was used to separate the proteins of interest, which were then transferred to a polyvinylidene difluoride (PVDF) membrane and soaked in a blocking solution [5% non-fat dry milk in TBST (tris-buffered saline, Tween 20)] for 1 h. The membrane was probed overnight with primary antibodies against WT1 (Abcam, Cambridge, UK), ErbB2 (Cell Signaling, Danvers, MA, USA), PDL1 (Santa Cruz Biotechnology, Santa Cruz, CA, USA), and β-Actin (Santa Cruz Biotechnology) at 4°C, and then incubated with horseradish peroxidase-conjugated goat anti-rabbit or anti-mouse polyclonal IgG secondary antibodies (Ab Frontier, Seoul, Republic of Korea). Chemiluminescent detection was performed using Immobilon Western Chemiluminescent HRP Substrate (Millipore Corporation, Billerica, MA, USA). β-Actin was used as an internal control. The expression of WT1, ErbB2, and PDL1 was determined using Amersham Imager 600 (GE Healthcare, Marlborough, MA, USA).

### Clonogenic Long-Term Survival Assay

Stable GL261 cells were harvested and irradiated at different doses (2, 4, and 6 Gy). Thereafter, GL261 cells (5 × 10^2^ cells/well) were reseeded in 6-well culture dishes and incubated at 37°C in an atmosphere of 5% CO_2_ for 14 d. The cells were fixed in methanol for 5 min and stained with toluidine blue (0.1%, Sigma, St. Louis, MO, USA) for 15 min. Dishes were washed with distilled water and dried at room temperature. Colony counting was performed on the following day. Colonies containing at least 50 cells were counted. The number of colonies in the irradiated wells was compared to the corresponding number in the non-irradiated wells. Plating efficiency was calculated as plating efficiency = [number of colonies counted/number of cells plated] ×100. Finally, the percentage survival fraction was calculated as survival fraction = [plating efficiency of treated sample/plating efficiency of control] ×100.

### MTT Assay

The effects of radiation on the proliferation of GL261 cells was estimated using the MTT assay. Briefly, after radiation with 2, 4, or 6 Gy, the cells (2.5 × 10^3^ cells/well) were seeded in 96-well plates and cultured with DMEM media supplemented with 10% FBS and 1% P/S at 37°C in an atmosphere of 5% CO_2_. Subsequently, the cells were stained every 24 h incubation until day 5 with 3-(4,5-dimethylthiazol-2-yl)-2,5-diphenyltetrazolium bromide (MTT; Sigma). For staining, the plates were washed with PBS, and MTT (0.5 mg/mL) was added to each well. The MTT solution was removed from each well after 4 h of incubation. MTT formazan was then solubilized using isopropanol (Merck, Darmstadt, Germany), and the optical density was read at 570 nm.

### Intracranial Glioma Mouse Model and Treatment Schedule

To establish the mouse intracranial model, we stereotactically injected 1 × 10^5^ GL261 cells in 5 μL PBS into the right striatum of the mice at a rate of 1 μL/min. Injection sites were estimated using the following coordinates: 2 mm anterior, 2 mm lateral from bregma, and 4 mm deep from the cortical surface (18). The mice was randomly allocated to the treatment arms. For treatment, the mice was divided into the following four treatment groups: (1) control; (2) RT only; (3) RT plus vaccine; and (4) RT plus vaccine and anti-PD1. On day 13 after injection, the mice were irradiated (6 Gy). Thereafter, branched multipeptide (150 μg/injection) and PADRE (50 μg/injection) were subcutaneously administrated on days 14 and 18. Poly-ICLC (Hiltonol, Oncovir Inc.) (50 μg/injection) was intramuscularly injected on the same day with peptide treatment (12, 19). The mice were also administered intraperitoneal injections of *in vivo* MAb anti-mouse PD1 (200 μg/injection) every other day (day 14, 16, and 18). Overall survival was quantified. The mice was euthanized on day 20 after injection to assess tumor size and immunological parameters in the spleen and tumor.

### Hematoxylin and Eosin (H&E) Staining of the Brain

Mouse brains were collected and fixed in formaldehyde. Thereafter, brains were sectioned into 4-mm thick slices at the injection site. Brain slices were stored in 5% paraformaldehyde, embedded in paraffin, and sectioned into 4-μm coronal sections using a microtome. For tumor size confirmation, H&E staining was performed. Briefly, hematoxylin was used to completely cover the tissue section in 5 min. After rinsing twice with distilled water to remove any excess stain, a bluing reagent was applied to the tissue for 1 min. Thereafter, slides were washed with distilled water and dipped in absolute alcohol. Finally, slides were incubated in eosin solution for 3 min, rinsed in distilled water, and dehydrated with absolute alcohol. Slides were then cleared and mounted using Histomount (National Diagnostics, USA). Tumor slides were scanned using the Aperio Scan Scope System (Aperio, Technology; Vista, CA, USA), and cross-section areas (mm^2^) of different treatment groups were confirmed using Aperio ImageScope software (Aperio). Data were summarized using bar charts.

### Isolation of Splenocytes and Single Tumor Cells

Splenocytes and single tumor cells were isolated directly from the spleen and tumor of non-vaccinated and vaccinated mice. For the isolation of splenocytes, the spleen was collected and washed with DMEM media supplemented with 10% FBS and 1% P/S. Then, a 1-mL syringe plunger was used to gently press the spleen through a 100-μm cell strainer (BD Falcon, Becton Dickinson, NJ, USA) while continuously adding media. After filtering through a 40-μm cell strainer (Falcon), erythrocytes were removed using 0.83% (w/v) NH_4_Cl (Sigma) (red blood cell lysis buffer). Cells were collected and washed with media. For the isolation of single tumor cells, the tumor was collected and washed with DMEM media supplemented with 10% FBS and 1% P/S. Subsequently, the tumor was minced into 3 to 4-mm pieces using a sterile scalpel. Tumor pieces were incubated with collagenase type IV (0.25%; Gibco-BRL) at 37°C in an atmosphere of 5% CO_2_ for 2 h. Samples were observed and suspended at 15-min intervals. Cells were filtered using 100- and 40-μm cell strainers (Falcon), and single tumor cells were collected. Erythrocytes were removed using the red blood cell lysis buffer.

### Flow Cytometry

For *in vitro* experiments, the expression of MHC I and PDL1 on GL261 cells before and after radiation was confirmed by flow cytometry. The cells were exposed to 2, 4, or 6 Gy radiation and cultured for the indicated time periods for flow cytometric analysis. Generally, the cells were stained with FITC-conjugated H-2Kb (BD Biosciences, San Jose, CA, USA) or PE-conjugated PDL1 (BD Biosciences) at the 0 and 24 h time points. Data were acquired on a BD FACS Calibur.

For the *in vivo* experiments, splenocytes, re-stimulated splenocytes, and tumor single cells were stained to confirm the immune cells. For cell surface staining, the cells (1 × 10^6^ cells) were stained with Pacific blue-conjugated CD45, PE-conjugated CD4 and CD8, PE-cy7-conjugated CD8, APC-conjugated CD44, APC-cy7-conjugated CD44, FITC-conjugated CD62L, FITC-conjugated CD69, PE-conjugated CD49b, FITC-conjugated CD279 (PD1), or PE-conjugated CD274 (PDL1) for 30 min at 4°C. For intracellular staining, the cells (1 × 10^6^ cells) were stained with PE-conjugated CD4, PE-cy7-conjugated CD8, or FITC-conjugated CD25 for 30 min at 4°C. The cells were then washed and permeabilized with FACS^TM^ Permeabilizing Solution 2 (BD Biosciences) for 30 min at room temperature. After washing twice with permeabilization buffer, the cells were stained with Alexa Fluor-conjugated Foxp3 or FITC-conjugated IFN-γ for 30 min at 4°C. For IFN-γ intracellular staining, the Protein Transport Inhibitor containing Brefeldin A (BD Golgi Plug^TM^) at 1 μL/1 × 10^6^ cell/well was added at the final *5* h of re-stimulation time. All antibodies were purchased from BD Biosciences. All samples were processed on a BD FACs Canto II (Becton Dickinson, Mountain View, CA, USA). Cell debris was eliminated by forward and side-scatter gating. All data were analyzed using FlowJo v10 software (TreeStar, San Carlos, CA, USA). Mean fluorescence intensity (MFI) ratio was calculated by dividing the median fluorescence intensity (MFI) of the positive cells (stained cell population) by that of the negative cells (unstained cell population).

### Splenocyte Re-stimulation and Single Tumor Cell Culture *ex vivo*

Splenocytes were re-stimulated according to the manufacturer's protocol. Splenocytes isolated from non-vaccinated and vaccinated mice after the final immunization (day 20) were cultured in 24-well plates (1 × 10^6^ cells/well) and re-stimulated with branched multipeptide (20 μg/mL) and PADRE (3 μg/mL) for 5 d in RPMI-1640 (Gibco-BRL) prepared in 10% FBS with 1% P/S supplementation and recombinant mouse (rm) IL-2 (20 ng/mL) (R&D systems). Anti-PD1 (10 μg/mL) was added during incubation. After re-stimulation, the supernatant and cells were collected and used for checking immune cell function. For IFN-γ intracellular staining, the splenocytes were re-stimulated and IFN-γ intracellular staining was performed or IFN-γ in the supernatant was estimated after 24-h incubation.

Single tumor cells from tumor were cultured in 6-well plates (1 × 10^6^ cells/well) for 24 h in 37°C in an atmosphere of 5% CO_2_, and the supernatant was collected for pro-inflammatory and anti-inflammatory cytokine determination by enzyme-linked immunosorbent assay (ELISA).

### IFN-γ Release Enzyme-Linked Immunospot (ELISPOT) Assay

The IFN-γ secreted by re-stimulated splenocytes against target cancer cells was examined using an IFN-γ ELISPOT assay kit (BD Biosciences). Ninety-six well PVDF membrane ELISPOT plates (Millipore, USA) were coated with the capture-purified anti-mouse IFN-γ antibody overnight at 4°C. Then, RPMI medium supplemented with 10% FBS was added to saturate the treated antibody. The re-stimulated splenocytes from the immunized mice were co-cultured with the target cells (GL261 and YAC-1 cell line) at a 10:1 ratio. Co-cultured cells were incubated in 10% FBS-RPMI medium for 24 h at 37°C in an atmosphere of 5% CO_2_. Subsequently, the plates were incubated for 2 h with the biotinylated detection anti-mouse IFN-γ antibody and then for 1 h with streptavidin-HRP. After washing, spots were revealed using an AEC substrate reagent set (BD Bioscience) and measured on an automatic CTL Immunospot Analyzer (Cellular Technology Ltd., Shaker Heights, OH, USA).

### Lactate Dehydrogenase (LDH) Release Cytotoxicity Assay

CytoTox 96 non-radioactive cytotoxicity assay (CytoTox 96, Promega, Madison, WI, USA) was performed to analyze the killing effects of the re-stimulated splenocyte effector cells against target cancer cells according to the manufacturer's instructions. GL261 and YAC-1 cell lines (2 × 10^5^ cells/well) were used as the target cells. The re-stimulated splenocytes were co-cultured with the target cells at a 10:1 ratio in Costar 96-well plates (Corning, Inc., Corning, NY, USA) for 4 h in 37°C and an atmosphere of 5% CO_2_. Then, supernatants were collected for determining the LDH concentration. The mean percentage of specific lysis was calculated as follows:

$$\%\text{Cyotoxicity}=\frac{\left(\text{Experimental - Effector Spontaneous - Target Spontaneous}\right)}{\left(\text{Target Maximum - Target Spontaneous}\right)}\times 100$$

### Enzyme-Linked Immunosorbent Assay (ELISA)

ELISA was performed to measure the levels of pro-inflammatory and anti-inflammatory cytokines released into the culture media of re-stimulated splenocytes or single tumor cells from non-vaccinated and vaccinated mice using the OptEIA ELISA set (BD Bioscience) following the manufacturer's instructions. Culture media from re-stimulated splenocytes isolated from vaccinated mice were analyzed for changes in the levels of the pro-inflammatory (IL-12p70 and IFN-γ) and anti-inflammatory (IL-10) cytokines, whereas the culture media of single tumor cells were analyzed for changes in the levels of the pro-inflammatory (IFN-γ) and anti-inflammatory [transforming growth factor-beta (TGF-β) and IL-10] cytokines.

### Statistical Analysis

All statistical analyses were performed using SPSS 20.0 for Windows (SPSS Inc., Chicago, IL, USA). One-way analysis of variance (ANOVA) was performed for analyses across multiple groups. The log-rank test was performed on survival data, and *p* < 0.05 was considered statistically significant. The results are represented as the means ± SD.

## Results

### Immunological Characteristics and Effects of Radiation on the GL261 Cell Line

To determine whether the tumor-associated antigens that were used for the synthesis of the branched multipeptide were indeed present on GL261 cells, we estimated the expression of the two tumor-associated antigens (ErbB2 and WT1) on GL261 cells. Our data indicated that GL261 cells have strong expression of both ErbB2 and WT1, and it is thus feasible to construct a branched multipeptide based on these antigens. Moreover, the high expression of MHC I and PDL1 on GL261 cells was also verified to confirm the efficiency of the treatment. Overall, GL261 cells showed immunological characteristics for immunotherapy.

**Figure 1:**
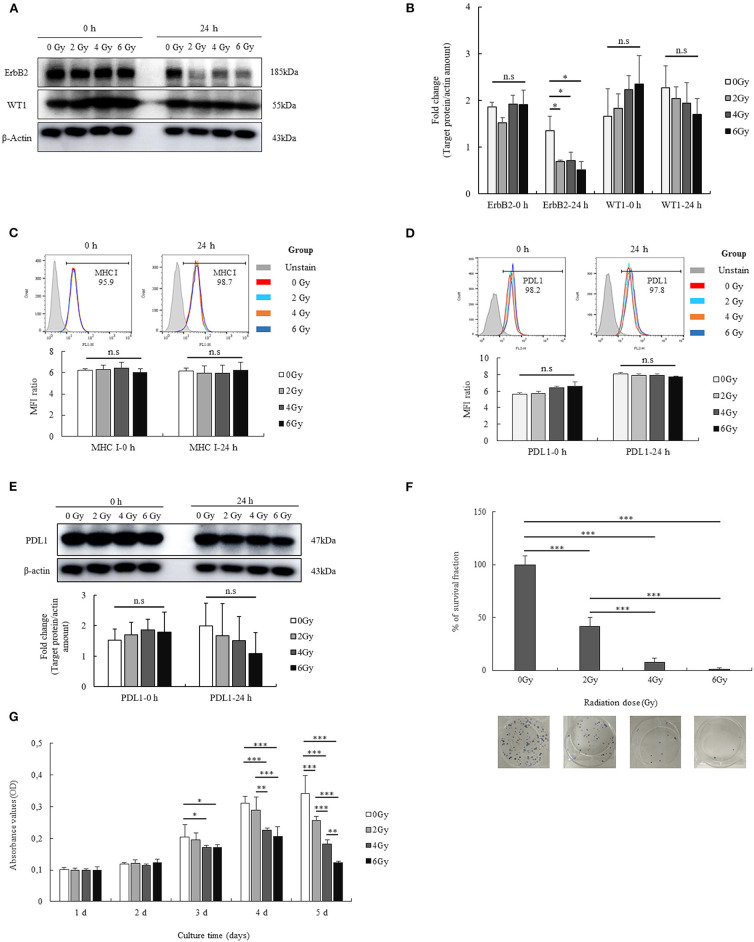
Immunological characterization of the GL261 cell line, and the effects of radiation. The expression of two tumor-associated antigens (ErbB2 and WT1) on GL261 cells and the short-term effects of radiation on the expression of these proteins were confirmed by western blot analysis **(A)**. Fold changes in protein signals are summarized by bar charts **(B)**. The expression of MHC I on GL261 cells and the short-term effects of radiation on this expression were also confirmed by flow cytometry **(C)**. The surface expression and total protein expression of PDL1 on GL261 was estimated by flow cytometry and western blot **(D,E)**. Moreover, the effects of radiation (2, 4, and 6 Gy) on the viability and proliferation of GL261 cells were also clarified by clonogenic assay and MTT assay **(F,G)**. Data is summarized by bar charts as the mean ± standard deviation (SD). All data are represented as the mean of two independent experiments. β-Actin was used as an internal control, and the figure is composed of multiple gel images. Full-length blots are presented in Figure S2. GL261, mouse glioblastoma cell line; H-2Kb, MHC class I expression in mice. *p < 0.05; **p < 0.01; and ***p < 0.001; n.s., no significant difference.

The short-term effects of radiation on GL261 cells were also determined. As shown in Figures 1A,B, the levels of ErbB2, WT1 expression were enhanced after treatment with the different radiation doses at 0 h and were reduced after 24 h. However, no differences were observed, except with regard to the expression of ErbB2. In particular, ErbB2 expression was reduced in all radiation groups treated with 2 Gy (*p* = 0.029), 4 Gy (*p* = 0.032), and 6 Gy (*p* = 0.013) compared with that in the no-radiation control group. Additionally, the effects of radiation on MHC I and PDL1 expression on GL261 cells was also confirmed at 0 and 24 h. The expression of MHC I and PDL1 on GL261 cells after irradiation (2, 4, and 6 Gy) is shown in Figures 1C–E; most GL261 cells showed strong surface expression of MHC I and PDL1 with no differences before and after radiation at 0 and 24 h, which paralleled the total PDL1 protein expression on GL261 cells. These results showed that the effects of radiation on GL261 cells after a short-term 24-h period only altered the expression of ErbB-2.

To investigate the long-term effects of radiation, we evaluated the viability and proliferation of GL261 cells after radiation with different doses (2, 4, and 6 Gy). The percentage survival fraction of GL261 cells after radiation was determined based on the results of the clonogenic assay. As shown in Figure 1F, GL261 cells showed reduced survival upon irradiation. Particularly, the survival fraction of GL261 cells was reduced to 58.17% with 2 Gy (*p* < 0.001), 92.37% with 4 Gy (*p* < 0.001), and 98.69% with 6 Gy (*p* < 0.001), compared with that of the no-radiation control. Moreover, the survival fraction of GL261 cells exposed to 4 and 6 Gy was also reduced compared with that of 2 Gy (34.2 and 40.5%, respectively, both *p* < 0.001). The effects of radiation on GL261 cell proliferation were also estimated. As shown in Figure 1G, irradiated GL261 cells showed a delayed proliferation as evidenced by the lower OD values in the MTT assay compared with that in the no-radiation control from day 3 after treatment. In particular, GL261 cells treated with 4 and 6 Gy radiation showed delayed proliferation compared with the no-radiation GL261 cells (*p* = 0.037 and *p* = 0.047, respectively) at day 3. Similarly, GL261 cells treated with 4 and 6 Gy radiation showed more delayed proliferation than the no-radiation GL261 cells (*p* = 0.000 and *p* = 0.000, respectively) on day 4. On day 5, GL261 cells treated with 2, 4, and 6 Gy radiation showed delayed proliferation compared with the no-radiation GL261 cells (*p* = 0.000, *p* = 0.000, and *p* = 0.000, respectively). Moreover, GL261 cells treated with 4 Gy radiation also had delayed proliferation compared with the 2 Gy-treated GL261 cells (*p* = 0.001), and GL261 cells treated with 6 Gy radiation had delayed proliferation compared with the 2 and 4 Gy-treated GL261 cells (*p* = 0.000 and *p* = 0.007, respectively). Overall, radiation affected the long-term survival and proliferation of GL261 cells *in vitro*.

### Therapeutic Effects of Radiation Combined With Vaccine and Anti-PD1 on the GBM Mouse Model

**Figure 2:**
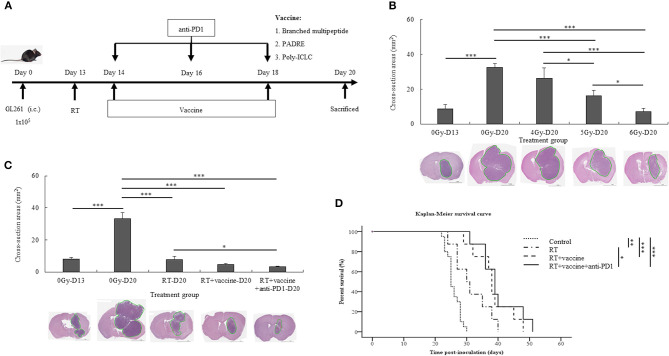
Experimental treatment schedule, and therapeutic effects of combination treatment. Schema outlining treatments used in the experiments **(A)**. Brain tumor size with and without radiation relative to the different radiation dose treatment (4, 5, and 6 Gy) was estimated with H&E staining **(B)**. The brain tumor sizes before (day 13) and after treatment (day 20) with different combinations, such as RT, RT plus vaccine, and RT plus vaccine and anti-PD1, were confirmed by H&E staining **(C)**. The Kaplan-Meier survival curves used to estimate the survival of the mouse tumor model according to the different treatment combinations in GL261 glioma-bearing mice, such as RT (*n* = 8), RT plus vaccine (*n* = 8), and RT plus vaccine and anti-PD1 (*n* = 8), compared with the untreated control (*n* = 20) **(D)**. Data are summarized by bar charts as the mean ± standard deviation (SD). All data are represented as the mean of two independent experiments. Control: no treatment group; RT, radiation therapy; vaccine, branched multipeptide plus PADRE and poly-ICLC; *p < 0.05; **p < 0.01; and ***p < 0.001.

The treatment schedule described in Figure 2A was followed. First, the radiation dose was screened in the mouse GBM model, and the optimal dose was selected for the subsequent *in vivo* experiments. On day 13, after transplantation, the mice were irradiated at different doses (4, 5, and 6 Gy). Thereafter, the mice were euthanized on day 20 to determine the tumor size; additionally, the tumor was also subjected to H&E staining. Tumor cross-sections at different radiation doses (4, 5, and 6 Gy) were studied (Figure 2B). In the 4 Gy-treated mice, no significant difference was noted in the cross-sectioned areas of the tumor compared to the control. However, the 5 and 6 Gy-treated mice showed smaller cross-sectioned areas than the no-radiation control (*p* = 0.001 and *p* = 0.000, respectively). Furthermore, 5 and 6 Gy-treated mice exhibited lower tumor sizes than the 4 Gy-treated mice (*p* = 0.014 and *p* = 0.000, respectively). Between 5 and 6 Gy-treated mice, the latter showed a greater effect on tumor proliferation that resulted in delayed tumor growth (*p* = 0.022). Radiation with 6 Gy on day 13 was chosen for the subsequent experiments.

After treatment with radiation plus vaccine and anti-PD1, the size of the brain tumor on day 20 and survival were investigated in the different treatment groups. The size of the brain tumor at the injection site before and after treatment was confirmed by H&E staining (Figure 2C). The tumor size before treatment was confirmed on day 13. On day 20, the tumor size was also compared between the control and treatment groups. A significant difference was noted in the cross-sectioned areas between the treatment groups and the control group. In particular, the control group showed larger cross-sectioned areas than in RT (*p* = 0.000), RT plus vaccine (*p* = 0.000), and RT plus vaccine and anti-PD1 (*p* = 0.000) groups. Although there was no significant difference between RT and RT plus vaccine or RT plus vaccine and RT plus vaccine and anti-PD1, RT plus vaccine and anti-PD1 showed smaller cross-sectioned areas than the RT group (*p* = 0.043). These data correspond with the results of mouse survival (Figure 2D). Mice exhibited prolonged survival following radiation treatment. In particular, RT enhanced survival from ~25.8 ± 2.2 days in the control to 31.5 ± 5.7 days in the RT group (*p* = 0.003), 38.3 ± 6.2 days in the RT plus vaccine (*p* < 0.001), and 40 ± 6.5 days in the RT plus vaccine and anti-PD1 group (*p* < 0.001). Although the RT plus vaccine and anti-PD1 group showed no significant difference from the RT plus vaccine group, the former exhibited prolonger survival than the RT only group (*p* = 0.022). Therefore, RT combined vaccine and anti-PD1 showed a prolonged mouse survival according to delay in tumor growth in GBM model.

### The Expression of PD1 on T Lymphocytes and That of PDL1 on Single Tumor Cells After Treatment

**Figure 3:**
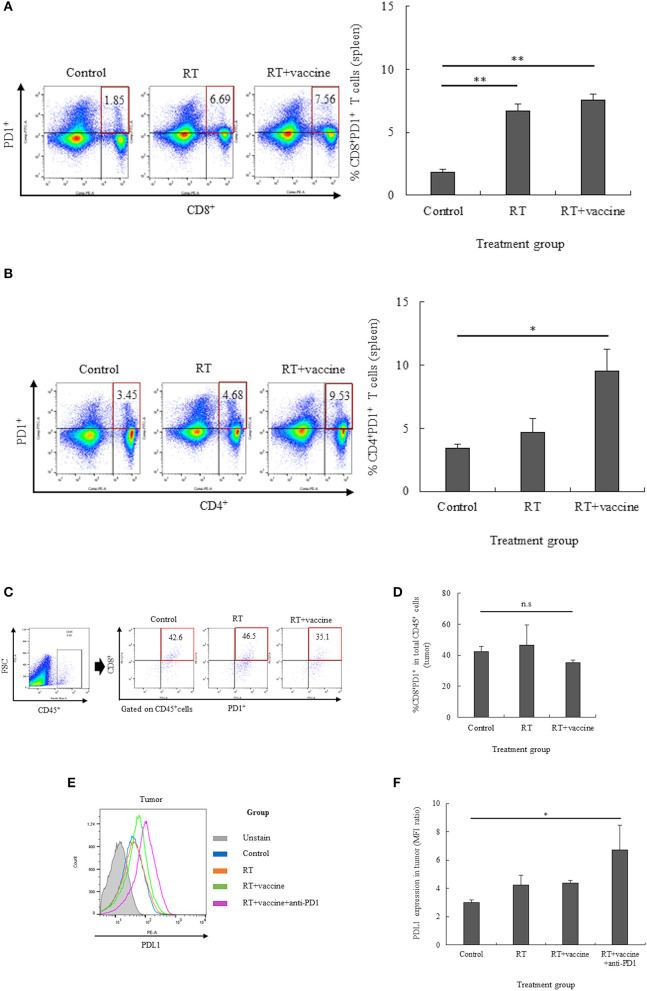
The expression of PD1 on T lymphocytes in the spleen and tumor and that of PDL1 on the tumor were estimated by flow cytometry. The expression of PD1 on the T lymphocyte population was estimated, and percentages of CD8^+^PD1^+^ T cells or CD4^+^PD1^+^ T cells in the spleen and CD8^+^PD1^+^ T cells in the tumor were determined **(A–D)**. The expression of PDL1 in the tumor was also clarified **(E,F)**. Data are summarized by bar charts as the mean ± standard deviation (SD). All data are represented as the mean of two independent experiments. Control, no treatment group; RT, radiation therapy; vaccine, branched multipeptide plus PADRE and poly-ICLC; *p < 0.05; **p < 0.01; and n.s., no significant difference.

The expression of the PD1 receptor on CD8^+^ and CD4^+^ T cells in the splenocytes and single tumor cells was confirmed by flow cytometry. The percentages of CD8^+^PD1^+^ cells and CD4^+^PD1^+^ cells in the splenocytes are shown in Figures 3A,B; RT only enhanced the expression of PD1 on CD8^+^ T cells, whereas RT plus vaccine enhanced the expression of PD1 on both CD8^+^ and CD4^+^ T cells. Notably, the percentage of CD8^+^PD1^+^ T cells was higher in the RT group (4.8%; *p* = 0.005) and in the RT plus vaccine group (5.7%; *p* = 0.003) than in the control group. The percentage of CD4^+^PD1^+^ T cells in the RT plus vaccine group was higher (by 6.08%) than that in the control group (*p* = 0.038). Moreover, the percentages of tumor-infiltrating CD8^+^PD1^+^ T cells on the single tumor cells were also confirmed. Although the RT plus vaccine group showed a slightly lower percentage of tumor-infiltrating CD8^+^PD1^+^ T cells than the control or RT only group, there was no difference between the RT plus vaccine group compared to RT only group or control group with regard to the single tumor cells (Figures 3C,D).

The expression of PDL1 on the single tumor cells was also estimated. As shown in Figures 3E,F, there was no significant difference between the control, RT only, and RT plus vaccine group. However, the addition of anti-PD1 to the RT plus vaccine group led greater PDL1 expression in the single tumor cells than in the control group (*p* = 0.049). Therefore, anti-PD1 may lead to an increase in the expression of PDL1 in the tumor.

### The Distribution of Immune Cells in the Splenocytes and Single Tumor Cells

**Figure 4:**
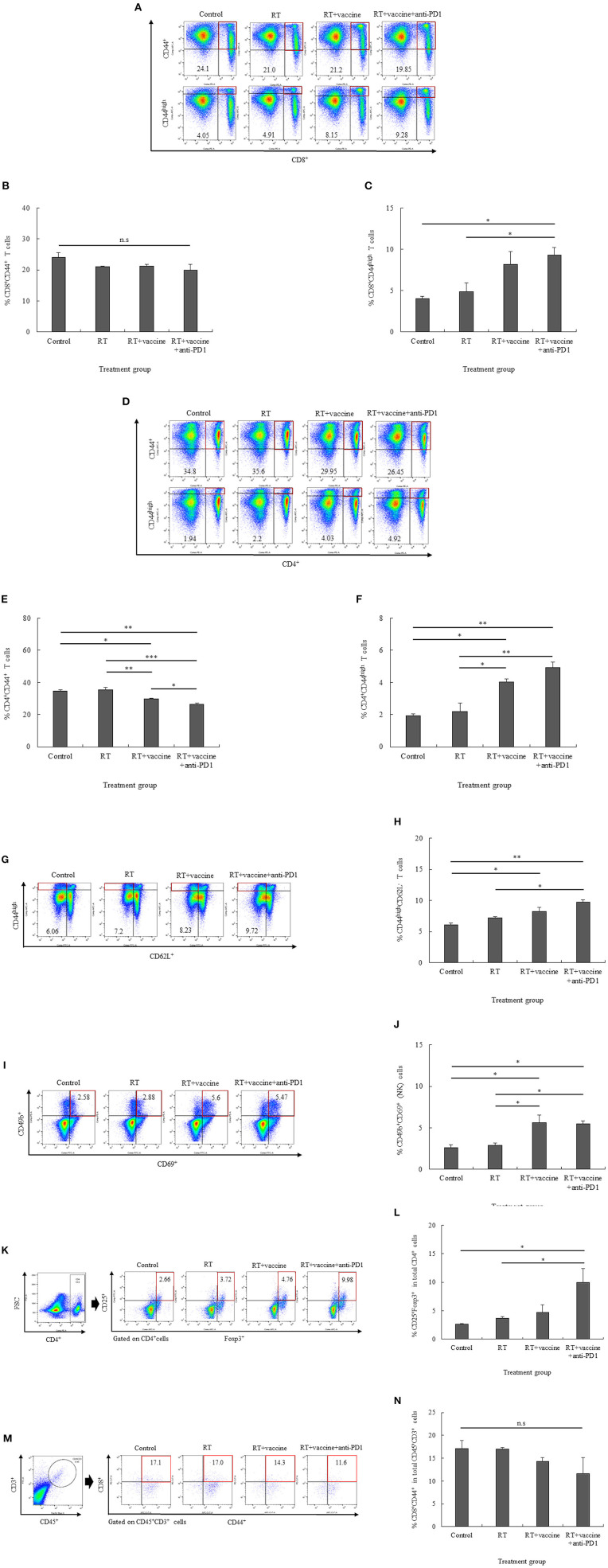
The distribution of immune cells in the spleen and tumor was confirmed by flow cytometry. The presentation of activated CD8^+^CD44^+^ T cells and high activated CD8^+^CD44^high^ effector T cells **(A–C)**, activated CD4^+^CD44^+^ T cells and high activated CD4^+^CD44^high^ effector T cells **(D–F)**, high activated CD44^high^CD62L^−^ effector memory T cells **(G,H)**, CD49b^+^CD69^+^ natural killer (NK) effector cells **(I,J)**, and CD4^+^CD25^+^Foxp3^+^ regulatory T cells **(K,L)** in the splenocytes of non-vaccinated and vaccinated mice was estimated. Moreover, the presentation of activated CD8^+^CD44^+^ T cells in the tumor was also confirmed **(M,N)**. Data are summarized by bar charts as the mean ± standard deviation (SD). All data are represented as the mean of two independent experiments. Control, no treatment group; RT, radiation therapy; vaccine, branched multipeptide plus PADRE and poly-ICLC; *p < 0.05; **p < 0.01; and ***p < 0.001; n.s., no significant difference.

In the splenocytes, both activated CD8^+^ and CD4^+^ T cells were enhanced in response to the combination treatment. Although the number of CD8^+^CD44^+^ T cells was not significantly different between the control and treatment groups, CD8^+^CD44^high^ T cells were increased in the RT plus vaccine and anti-PD1 group (Figures 4A–C). In particular, the RT plus vaccine and anti-PD1 group exhibited a higher percentage of CD8^+^CD44^high^ T cells compared with that in the control (5.2%, *p* = 0.025) and the RT only group (4.37%, *p* = 0.043). Similarly, CD4^+^CD44^+^ T cells showed a reduction in response to the RT plus vaccine or RT plus vaccine and anti-PD1 treatments, whereas CD4^+^CD44^high^ T cells showed an increase in response to these treatments (Figures 4D–F). In particular, RT plus vaccine reduced the percentage of CD4^+^CD44^+^ T cells compared with that in the control (4.85%, *p* = 0.012) and the RT only group (5.65%, *p* = 0.007). Moreover, RT plus vaccine and anti-PD1 reduced CD4^+^CD44^+^ T cells compared with that in the control (8.35%, *p* = 0.002), the RT only (9.15%, *p* = 0.001), and the RT plus vaccine group (3.5%, *p* = 0.034). However, RT plus vaccine enhanced CD4^+^CD44^high^ T cells compared with that in the control group (2.1%, *p* = 0.011) and the RT only group (1.63%, *p* = 0.017). In addition, RT plus vaccine and anti-PD1 enhanced CD4^+^CD44^high^ T cells compared with that in the control group (2.98%, *p* = 0.003) and the RT only group (2.52%, *p* = 0.004).

Moreover, RT plus vaccine or RT plus vaccine and anti-PD1 also enhanced the effector memory T cell counts in the splenocytes (Figures 4G,H). In particular, the RT plus vaccine group has a higher percentages of CD44^high^CD62L^−^ T cells than the control group (2.17%; *p* = 0.021), whereas the RT plus vaccine and anti-PD1 group had a higher percentage of CD44^high^CD62L^−^ T cells than the control group (3.66%; *p* = 0.003) and the RT only group (2.52%; *p* = 0.013). Similarly, the percentages of activated NK cells (CD49b^+^CD69^+^) in the splenocytes also showed an increase in the RT plus vaccine or RT plus vaccine and anti-PD1 groups (Figures 4I,J). In particular, the RT plus vaccine group had higher percentages of CD69^+^CD49b^+^ NK cells compared with that in the control (3.02%; *p* = 0.018) and the RT only group (2.72%; *p* = 0.026). Moreover, RT plus vaccine and anti-PD1 group had higher percentages of CD69^+^CD49b^+^ NK cell levels than the control (2.89%; *p* = 0.021) and the RT only group (2.6%; *p* = 0.03).

The treatment groups showed increased percentages of not only activated T cells and NK cells but also regulatory T cells (Tregs) in the splenocytes. The addition of anti-PD1 to the RT plus vaccine group resulted in an increased percentage of CD4^+^CD25^+^Foxp3^+^ Tregs (Figures 4K,L). Notably, RT plus vaccine and anti-PD1 treatment lead to higher CD4^+^CD25^+^Foxp3^+^ Treg cell counts than the control group (7.32%; *p* = 0.02) and the RT only group (6.26%; *p* = 0.033).

The percentages of tumor-infiltrating CD8^+^CD44^+^ T cells were also confirmed by flow cytometry. As shown in Figures 4M,N, there was a small amount of tumor-infiltrating CD45^+^CD3^+^ cells were detected and no significant difference between the tumor-infiltrating CD8^+^CD44^+^ cells gated from CD45^+^CD3^+^ cells in all the treatment groups compared with the control group. Moreover, tumor-infiltrating CD4^+^CD44^+^ cells gated from CD45^+^CD3^+^ cells were not detected in our study (data not shown).

### Pro-inflammatory and Anti-inflammatory Cytokine Production From Re-stimulated Splenocytes

**Figure 5:**
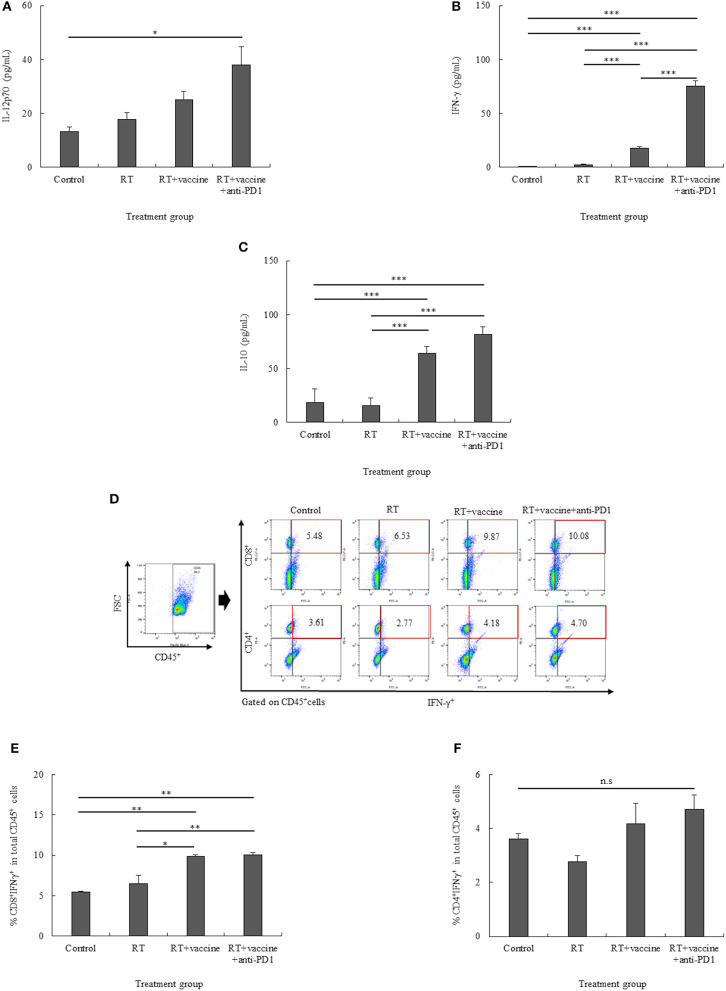
The pro-inflammatory (IL-12p70 and IFN-γ) and anti-inflammatory (IL-10) cytokine levels in the re-stimulated splenocytes were determined using ELISA **(A–C)**. The percentages of CD8^+^IFN-γ^+^ cells and CD4^+^IFN-γ^+^ cells in the re-stimulated splenocytes were also confirmed **(D–F)**. Data are summarized by bar charts as the mean ± standard deviation (SD). All data are represented as the mean of two independent experiments with total 5 mouse per group. Control, no treatment group; RT, radiation therapy; vaccine, branched multipeptide plus PADRE and poly-ICLC; *p < 0.05; **p < 0.01; and ***p < 0.001.

For pro-inflammatory cytokines, IL-12p70 and IFN-γ were investigated. There was an increase in the IL-12p70 levels in response to the treatments; however, there was no significant difference between the treatments, except for the RT plus vaccine and anti-PD1 group (Figure 5A). In particular, the RT plus vaccine and anti-PD1 group showed a significant difference in the IL-12p70 levels compared with the control group (*p* = 0.044). With regard to IFN-γ, only the RT group showed no significant difference compared with the control; the RT plus vaccine or RT plus vaccine and anti-PD1 groups showed increase compared with the control and RT group (Figure 5B). In particular, RT plus vaccine group exhibited higher IFN-γ levels than the control (*p* < 0.001) and RT groups (*p* < 0.001), whereas the RT plus vaccine and anti-PD1 group showed the highest levels compared with the control (*p* < 0.001), RT (*p* < 0.001), and RT plus vaccine groups (*p* < 0.001). The anti-inflammatory cytokine IL-10 levels were enhanced in response to treatment (Figure 5C). Although RT alone showed no significant difference compared with the control group in terms of IL-10 levels, the RT plus vaccine or RT plus vaccine and anti-PD1 groups showed higher IL-10 levels than the control and RT groups. In particular, the RT plus vaccine group showed higher IL-10 levels than the control (*p* < 0.001) and RT only groups (*p* < 0.001). Moreover, the RT plus vaccine and anti-PD1 group had higher IL-10 production than the control (*p* < 0.001) and RT only groups (*p* < 0.001).

The expression of IFN-γ on CD8^+^ and CD4^+^ T cells was also clarified. As shown in Figures 5D–F, the percentages of CD8^+^IFN-γ^+^ cells were higher in the RT plus vaccine or RT plus vaccine and anti-PD1 groups than in the control and RT only groups. Particularly, RT plus vaccine enhanced compared with the control group (4.39%, *p* = 0.004) and the RT only group (3.34%, *p* = 0.012). Similarly, RT plus vaccine and anti-PD1 increased compared the control group (4.6%, *p* = 0.004) and the RT only group (3.55%, *p* = 0.009). There was no difference between the RT only and control or RT plus vaccine and RT plus vaccine and anti-PD1 groups. In contrast, although there was a slightly higher percentage of CD4^+^IFN-γ^+^ cells in the RT plus vaccine with or without anti-PD1 groups than in the RT only or control groups, there was no significant difference between all the treatment groups was found. The IFN-γ released in the supernatant after blocking for IFN-γ intracellular staining was also examined; IFN-γ in supernatant showed low levels, and no significant differences between the treatment groups were detected after blocking before intracellular staining (Figure S3). Our data showed that the RT plus vaccine and anti-PD1 group exhibited the pro-inflammatory cytokine IFN-γ mainly secreted by CD8^+^ cells in the re-stimulated splenocytes, which play an important role in stimulating the immune response.

### CTL and NK Cell Function of the Re-stimulated Splenocytes

**Figure 6:**
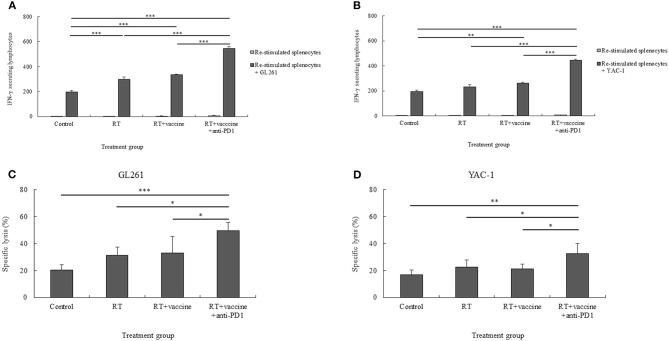
The cytotoxic T lymphocyte (CTL)- and natural killer (NK) cell-mediated immune response of re-stimulated splenocytes from vaccinated mice. The IFN-γ secreted by re-stimulated splenocytes when co-cultured with target cancer cells was measured using the IFN-γ ELISPOT assay **(A,B)**. The specific killing effects of re-stimulated splenocytes against target cancer cells was confirmed using the LDH assay **(C,D)**. The GL261 and YAC-1 cell lines were used as target cells for CTL and NK cell sensitivity, respectively. All data are represented as the mean of two independent experiments. GL261, mouse glioblastoma cell line; YAC-1, mouse lymphoma cell lines sensitive to the cytotoxic activity of naturally occurring killer cells in mice; Control, no treatment group; RT, radiation therapy; vaccine, branched multipeptide plus PADRE and poly-ICLC; *p < 0.05; **p < 0.01; and ***p < 0.001.

The CTL- and NK cell-mediated immune responses of the re-stimulated splenocytes from non-vaccinated and vaccinated mice were elucidated. IFN-γ secretion by the re-stimulated splenocytes after co-culture with target cancer cells was investigated for the anti-tumor effect of combination treatment in a murine GBM model. Re-stimulated splenocytes from non-treated and treated mice were prepared for IFN-γ ELISPOT assays. GL261 and YAC-1 cells were used as target cancer cells for investigating the CTL and NK cell activity, respectively. As shown in Figures 6A,B, mice treated with RT only showed an enhanced level of IFN-γ-secreting splenocytes against GL261 target cells whereas the RT plus vaccine- or RT plus vaccine and anti-PD1-treated mice showed an increase in the IFN-γ-secreting splenocytes against both GL261 and YAC-1 target cells. In particular, the RT plus vaccine group showed a higher level of IFN-γ-secreting splenocytes against GL261 and YAC-1 cells than the control (*p* < 0.001 and *p* = 0.003, respectively). Moreover, the RT plus vaccine and anti-PD1 group showed a higher level of IFN-γ-secreting splenocytes against GL261 and YAC-1 target cells than the control, RT only, and RT plus vaccine (all *p* < 0.001). However, there was no significant difference between the RT only and the RT plus vaccine groups with regard to the IFN-γ-secreting splenocytes.

The specific lysis of the re-stimulated splenocytes against target cancer cells was also confirmed. As shown in Figures 6C,D, RT plus vaccine and anti-PD1 enhanced the specific lysis of the re-stimulated splenocytes against both GL261 and YAC-1 cells. Particularly, with GL261 target cells, the RT plus vaccine and anti-PD1 group showed higher specific lysis than the control group (29%, *p* = 0.001), the RT only group (18.28%, *p* = 0.013), and the RT plus vaccine group (16.31%, *p* = 0.023). Similarly, with YAC-1 target cells, the RT plus vaccine and anti-PD1 group showed higher specific lysis than the control group (15.68%, *p* = 0.003), the RT only group (9.84%, *p* = 0.038), and the RT plus vaccine group (11.2%, *p* = 0.021). There was no significant difference in the percentages of specific lysis between the RT only or RT plus vaccine groups compared with the control group against GL261 and YAC-1 cells.

Although there was a higher number of IFN-γ-secreting splenocytes in the RT only, RT plus vaccine, or RT plus vaccine and anti-PD1 groups than in the control group, only the RT plus vaccine and anti-PD1 group showed highest percentages of specific lysis against GL261 and YAC-1 target cells than the control group. Moreover, these data showed an enhanced function in both CTL and NK cell activity. However, CTL activity in the re-stimulated splenocytes showed a greater shift than NK activity (Figures S4A,B). In particular, with IFN-γ-secreting splenocytes, the CTL activity was higher than the NK activity in the RT group (*p* = 0.029), RT plus vaccine group (*p* = 0.000), and RT plus vaccine and anti-PD1 group (*p* = 0.002). Similarly, the percentage of specific lysis of re-stimulated splenocytes against GL261 cells was also higher than that against YAC-1 cells in the RT plus vaccine and anti-PD1 group (*p* = 0.003).

### Pro-inflammatory and Anti-inflammatory Cytokines From the Single Tumor Cells

**Figure 7:**
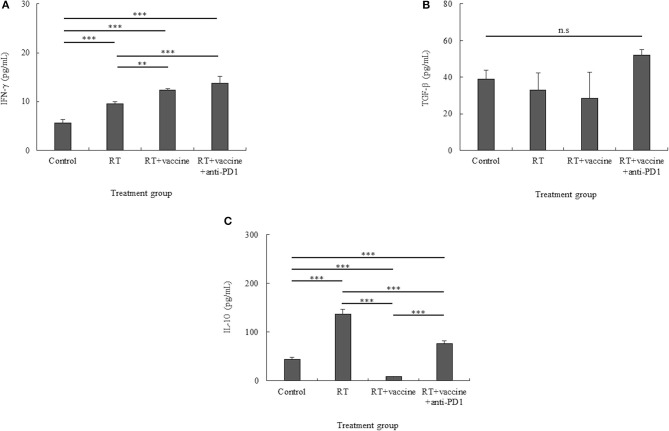
Pro-inflammatory and anti-inflammatory cytokine production in the culture media of single tumor cells was confirmed using ELISA. Pro-inflammatory cytokines such as IFN-γ **(A)** and anti-inflammatory cytokines such as IL-10 and TGF-β **(B,C)** were identified. While IFN-γ showed an increase, TGF-β was stable in all the treatment groups, and IL-10 showed different levels according to the different treatments. Data are summarized by bar charts as the mean ± standard deviation (SD). All data are represented as the mean of two independent experiments. Control, no treatment group; RT, radiation; vaccine, branched multipeptide plus PADRE and poly-ICLC; **p < 0.01; and ***p < 0.001; n.s., no significant difference.

IL-10 and TFG-β have been identified as key factors that mediate inhibitory action, whereas IFN-γ has been shown to be a pro-inflammatory cytokine in tumors. Culture media from single tumor cells during 24-h incubation were used to estimate the levels of the cytokines IFN-γ, IL-10, and TFG-β. As shown in Figure 7A, although single tumor cells present low levels of IFN-γ, these levels were higher in all the treatment groups than in the control. In particular, the RT only group had higher levels than the control (*p* < 0.001). Moreover, the RT plus vaccine group had higher IFN-γ levels than the control (*p* < 0.001) and RT only (*p* = 0.002) groups. Although RT plus vaccine and anti-PD1 showed higher IFN-γ levels than the control (*p* < 0.001) and RT plus vaccine (*p* < 0.001) groups, there was no difference between the RT plus vaccine and RT plus vaccine and anti-PD1 groups.

While TGF-β levels showed no difference between all treatment groups and the control, IL-10 showed different cytokine levels according to the various treatments administered. In particular, although TGF-β was increased in the RT plus vaccine and anti-PD1 group, there was no significant difference between the RT plus vaccine and anti-PD1 group compared with other groups (Figure 7B). In Figure 7C, whereas the IL-10 level was higher in the RT only group than in the control group (*p* < 0.001); the RT plus vaccine group showed lower levels of IL-10 than the control (*p* < 0.001) and RT only (*p* < 0.001) groups. Moreover, adding anti-PD1 to the RT plus vaccine group led to the recovery of the IL-10 levels in the single tumor cells. However, this IL-10 level was lower than that observed with RT only (*p* < 0.001). Therefore, combination treatment with RT along with the vaccine and anti-PD1 showed potential to produce pro-inflammatory response in the tumor.

## Discussion

ErbB2 and WT1 are not only tumor associated antigens for immunological targeting (20, 21) but also biomarkers of cancer cell proliferation and survival, especially GBM (22–25). In this study, a branched multipeptide was synthesized on the basis of the high expression of tumor-associated antigens on GL261 cells. Moreover, RT also showed short- and long-term effects on the GL261 cell line *in vitro*. In particular, radiation affects GL261 cells by reducing ErbB2 expression after a short (24 h) period as evidenced by the decrease in the long-term survival and proliferation of GL261 cells *in vitro*. These data showed that GL261 cells have both immunological characteristics and radiosensitive activity. Moreover, radiation also resulted in delayed tumor growth in the mouse model. This was expected, since the main therapeutic function is from radiation during early treatment stages. After a 24-h period, radiation starts to cause a delay in tumor proliferation and tumor cell death, resulting in the release of tumor-associated antigens for further immune response stimulation. The combination of RT with vaccines may bring about optimal results to further enhance the immune response in later treatment stages, when cancer cells recover and function normally.

The effectiveness of immune checkpoint blockade is hypothesized to require the expression of PDL1 on tumor cells and PD1 on peritumoral CTLs (26). In the present study, the expression of PD1 on T cell populations from splenocytes and single tumor cells and that of PDL1 on glioblastoma target cells and single tumor cells were confirmed to verify the efficiency of anti-PD1 treatment. Previous studies showed that the presence of the cytokine IFN-γ leads to enhanced PDL1 expression on tumors as a mechanism by which cancer cells protect themselves from T cell-mediated destruction (27–29). In the present study, anti-PD1 combined with RT plus vaccine also enhanced the levels of IFN-γ, which led to enhanced PDL1 expression in single tumor cells. Although our data showed enhanced IFN-γ levels in single tumor cells, this level was quite low; we are yet to clarify the source of the IFN-γ released by the tumor-infiltrating immune cells. The percentages of tumor-infiltrating CD45^+^CD3^+^ cells as well as tumor-infiltrating CD8^+^CD44^+^ T cells gated from CD45^+^CD3^+^ cells were quite low in this study. There was no difference in the percentages of tumor-infiltrating CD8^+^CD44^+^ T cells observed between the different treatment groups, and no tumor-infiltrating CD4^+^CD44^+^ T cells gated from CD45^+^CD3^+^ cells were detected in our study (data not shown). The distribution of lymphoid lineage cells in lymph nodes and myeloid lineage cells was not clarified in this study. Therefore, these immune cells should be investigated in greater detail in future studies.

While PD1 blockade enhanced the cytotoxic efficacy of CD8^+^ CTLs, it also enhanced the proliferation and immunosuppressive activity of Tregs in humans and mice (30, 31). Similarly, in our study, the addition of anti-PD1 into the RT plus vaccine group also led to an increase in both the activated effector cells (CD8^+^ T cells, CD4^+^ T cells, NK cells, and memory T cells) and suppressor immune cells (Tregs) in the splenocytes. Although the percentages of Tregs were also enhanced by the addition of anti-PD1 in the RT plus vaccine group, RT plus vaccine and anti-PD1 therapy still showed a shift to effector immune cell function. In particular, the RT plus vaccine and anti-PD1 group showed the highest CTL and NK cell-mediated tumor cell-targeting immune response following prolonged mouse survival compared with the other treatment groups. In contrast, dendritic cells were responsible for the uptake of tumor-associated antigens from the treated peptide vaccine or dying tumor cells induced by radiation. This led to DC maturation, which stimulated both the innate and adaptive immune systems. Mature DCs not only activate CTLs to target tumors but are also capable of activating NK cells by enhancing their cytotoxicity, IFN-γ production, and the crosstalk of NK cells; DCs also play an important role in the induction of the tumor-specific immune response against cancer (32, 33). Although our data showed an increase in both CTL and NK cell function targeting tumor cells, our results mainly support a shift in CTL activity rather than NK activity.

Cytokines play an important role in mediating and regulating the immune response. Examining both pro-inflammatory and anti-inflammatory cytokine levels is important while verifying cancer treatment effects (34, 35). In our data, although there were high levels of pro-inflammatory cytokines in the re-stimulated splenocytes (IL-12p70 and IFN-γ) and single tumor cells (IFN-γ), the levels of anti-inflammatory cytokines (IL-10) in the re-stimulated splenocytes and single tumor cells were also increased. We found that the IFN-γ released in the re-stimulated splenocytes mainly originated from CD8^+^ T cells. Particularly, the increase in IL-10 paralleled the increased percentage of Tregs in the re-stimulated splenocytes. Both TGF-β and IL-10 cytokines are known to be suppressive cytokines mainly released by Tregs, which may directly suppress effector T cells in the tumor microenvironment (36). While no difference in TGF-β was observed between all the treatment groups, IL-10 showed an increased level in single tumor cells. Similar patterns may occur in single tumor cells, whereby enhanced IL-10 increases the percentage of Tregs. However, Tregs in single tumor cells were not examined in our study. This supports the notion that enhanced Tregs may be related to enhanced IL-10, which subsequently resulted in no significant difference between RT plus vaccine and RT plus vaccine and anti-PD1. However, more experiments should be conducted to clarify this issue.

It is well-known that GBM has an unfavorable prognosis, mainly owing to its high propensity for tumor recurrence: more than 90% of patients show recurrence at the original tumor location and 5% develop multiple lesions after treatment (37). Enhanced effector memory T cell with RT plus vaccine and anti-PD1 have the potential to prevent GBM recurrence after treatment. RT plus vaccine and anti-PD1 is preferable in GBM treatment. However, the results of our study did not fully elucidate the exact manner in which this combination affected glioblastoma recurrence.

## Conclusion

In this study, a branched multipeptide and adjuvants, such as PADRE and poly-ICLC, were used as components of a vaccine. Our study suggests that immunotherapy using this vaccine combined with anti-PD1 could be helpful for improving RT effects in a GBM mouse model.

## Data Availability Statement

The datasets generated in this study are available on request from the corresponding author.

## Ethics Statement

This animal study was reviewed and approved by Chonnam National University Animal Research Committee.

## Author Contributions

T-A-TT, Y-HK, and T-YJ designed and performed the experiment. T-A-TT, Y-HK, and T-H-OD analyzed the data. T-A-TT and T-YJ wrote the article. SJ, I-YK, K-SM, W-YJ, H-JL, J-JL, and T-YJ contributed intellectually to the research.

## Conflict of Interest

The authors declare that the research was conducted in the absence of any commercial or financial relationships that could be construed as a potential conflict of interest.

## Supplementary Material

Figure S1 Construction of the branched multipeptide. The branched multipeptide was designed by combining two single peptides (ErbB2_aa63−71_ and WT1_aa235−243_) that were restricted to H-2b. Mini-polyethylene glycol (mini-PEGs) spacers were used to create the corresponding branched multipeptide by incorporating two single MHC I peptides. These were designated as CYTWNQMNL-miniPEG2-K (TYLPANASL-miniPEG2) for the *in vivo* experiment.

Figure S2 Specific antibodies against ErbB2, WT1, and PDL1 in the mouse GL261 cell line. Protein marker molecular weights are given in kDa. Full gel expression of ErbB2, WT1, and PDL1 against mouse GL261 cells was confirmed by western blot analysis **(A–D)**. Changes in the expression of these proteins before and after radiation with 2, 4, and 6 Gy was confirmed at 0 and 24 h. The estimated band was cut and presented **(E,F)**. β-Actin was used as an internal control in all the western blot experiments. GL261, mouse glioblastoma cell line.

Figure S3 IFN-γ in the supernatant after membrane blocking for IFN-γ intracellular staining was confirmed using ELISA. Data are summarized by bar charts as the mean ± standard deviation (SD). All data are represented as the mean of two independent experiments. Control: no treatment group; RT: radiation; vaccine: branched multipeptide plus PADRE and poly-ICLC; n.s., no significant difference.

Figure S4 The cytotoxic T lymphocyte (CTL)- and natural killer (NK) cell-mediated immune response of re-stimulated splenocytes from vaccinated mice. The IFN-γ secreted by re-stimulated splenocytes when co-cultured with target cancer cells was measured using the IFN-γ ELISPOT assay **(A)**, and the specific killing effects of re-stimulated splenocytes against target cells was estimated by the LDH assay **(B)**. The GL261 and YAC-1 cell lines were used as target cells for CTL and NK cell sensitivity, respectively. All data are represented as the mean of two independent experiments. Control, no treatment group; GL261, mouse glioblastoma cell line; YAC-1, mouse lymphoma cell lines sensitive to the cytotoxic activity of naturally occurring killer cells in mice; RT, radiation therapy; vaccine, branched multipeptide plus PADRE and poly-ICLC; ^*^*p* < 0.05; ^**^*p* < 0.01; and ^***^*p* < 0.001.
